# WordSeeker: concurrent bioinformatics software for discovering genome-wide patterns and word-based genomic signatures

**DOI:** 10.1186/1471-2105-11-S12-S6

**Published:** 2010-12-21

**Authors:** Jens Lichtenberg, Kyle Kurz, Xiaoyu Liang, Rami Al-ouran, Lev Neiman, Lee J Nau, Joshua D Welch, Edwin Jacox, Thomas Bitterman, Klaus Ecker, Laura Elnitski, Frank Drews, Stephen Sauchi Lee, Lonnie R Welch

**Affiliations:** 1 Bioinformatics Laboratory, School of EECS, Ohio University, Athens, Ohio 45701, USA; 2 Developmental Biology Institute of Marseille, Luminy F-13009, Marseille, France; 3 Cyberinfrastructure Group, Ohio Supercomputer Center, Columbus, Ohio 43212, USA; 4 Genomic Functional Analysis Section, National Human Genome Research Institute, NIH, Rockville, Maryland 20892 USA; 5 Department of Statistics, University of Idaho, Moscow, Idaho 83844, USA; 6 Biomedical Engineering Program, Ohio University, Athens, Ohio 45701, USA; 7 Molecular and Cellular Biology Program, Ohio University, Athens, Ohio 45701, USA

## Abstract

**Background:**

An important focus of genomic science is the discovery and characterization of all functional elements within genomes. *In silico* methods are used in genome studies to discover putative regulatory genomic elements (called words or motifs). Although a number of methods have been developed for motif discovery, most of them lack the scalability needed to analyze large genomic data sets.

**Methods:**

This manuscript presents WordSeeker, an enumerative motif discovery toolkit that utilizes multi-core and distributed computational platforms to enable scalable analysis of genomic data. A controller task coordinates activities of worker nodes, each of which (1) enumerates a subset of the DNA *word space* and (2) scores words with a distributed Markov chain model.

**Results:**

A comprehensive suite of performance tests was conducted to demonstrate the performance, speedup and efficiency of WordSeeker. The scalability of the toolkit enabled the analysis of the entire genome of *Arabidopsis thaliana*; the results of the analysis were integrated into The Arabidopsis Gene Regulatory Information Server (AGRIS). A public version of WordSeeker was deployed on the Glenn cluster at the Ohio Supercomputer Center.

**Conclusion:**

WordSeeker effectively utilizes concurrent computing platforms to enable the identification of putative functional elements in genomic data sets. This capability facilitates the analysis of the large quantity of sequenced genomic data.

## Background

The importance of discovering the patterns and features in genomic sequences is motivated by a number of scientific contexts. The Encyclopedia of DNA Elements project (ENCODE) seeks ‘to identify all functional elements in the human genome sequence’ [1]. Another context, the study of co-regulated genes, involves the analysis of the promoter sequences, introns, and untranslated regions (UTRs) of genes that were determined by microarray experiments to be co-regulated. Similarly, transcription factor binding regions identified by ChIP-chip and ChIP-Seq experiments are examined to identify genomic patterns [2]. Genome-wide pattern discovery studies, which seek to identify vocabularies of genomes [3,4], provide yet another perspective on genomic data. Large scale analysis of genomic data is also performed in the search for genomic signatures (unique elements that characterize specific organisms, tissues, pathways, and functions) [5]. All of these problems require the discovery of patterns in genomic sequences.

Several approaches have been developed for genomic pattern discovery. Word enumeration methods are algorithmic techniques that systematically discover either substrings (i.e., *words*) or sets of related substrings (i.e., *motifs*) in DNA sequences. Most enumeration methods create a data representation of the input sequence(s) that provides fast retrieval of elementary word statistics. The representation serves as a central data structure for a number of other analyses, including statistical word scoring, word-clustering, and motif discovery. A number of algorithmic techniques for word space enumeration have been proposed. Each of the enumeration algorithms can be classified as either *index-based*[6-19], *graph-based*[20], or *iterative*[21,22].

*Index-based* enumeration strategies create a data structure, called the index, and provide a mapping function, which maps the character composition of a word to a specific entry in the index data structure. Index-based strategies differ in (1) the data structure used for the index and (2) the type of mapping function employed. Popular index-based strategies employ hash functions, radix trees, and suffix trees. *YMF*[6,13,14], *Wordspy*[15,16], and *RMES*[8,9,11] employ *hash functions* for enumerating the word space. An alternative to hash functions, *radix trees* require *O(n^2^)* space (where *n* is the total number of characters in the input sequences), and are among the fastest representations for the retrieval of words. *Seeder*[7] and *SMS*[12] are examples of approaches that utilize a *radix tree* for storing words. A third alternative for index data structure, *suffix trees* provide a semantically rich representation of a set of input sequences. They require *O(n)* time and space, and enable a number of efficient and elegant string processing algorithms. Many tools and algorithms based employ suffix trees, including *Speller*[10], *Weeder*[17], *REPuter*[18], and *Verbumculus*[19].

*Winnower*[20], a *graph-based* approach, has been used for solving *the Planted (l,d) Motif* problem [20] (the problem of finding a motif of length *l* occurring among all sequences in a set, allowing for at most *d* mismatches between the instances of the motif). The Winnower algorithm reduces the problem of finding (l, d) motifs to the problem of finding large cliques in multi-partite graphs. The undirected Winnower graph *G* contains nodes representing words, and edges representing a similarity relationship (e.g., hamming distance) between words. Instead of finding maximal cliques, which is an NP-complete problem [23], Pevzner and Sze iteratively remove edges from *G* that are guaranteed not to be contained in a clique of size *k*, resulting in an algorithm of O(N^k+1^), where *N* is the total number of nucleotides.

*Iterative* approaches, such as *Teiresias*[22] and *Mitra*[21], incrementally concatenate short motifs from the input sequences to discover maximal motifs. These methods generate the set of maximal patterns without having to enumerate the entire word-space of an input sequence set. The Teiresias algorithm divides the motif discovery process into two phases: scanning and convolution. During the scanning phase, a set of elementary patterns of length *W*, satisfying a user-defined quorum *q*, is enumerated for a specific length with a required number of non-mismatches *L*. During the convolution phase, the elementary patterns are combined pair-wise and the resulting patterns are added to the set of elementary patterns if they satisfy the quorum. During convolution it is necessary to consistently detect and remove patterns that are no longer maximal, but are instead part of larger patterns with the same quorum satisfaction. The complexity of the scanning phase is O(NW^L^), with N being the total number of nucleotides, and the complexity of the convolution phase is , (where *rc(T’)* represents the matches in a pattern *T’*, which is a maximization of a pattern *T*[24]). Taking into consideration all calls to a maximization function, the worst-case time complexity of the Teiresias algorithm is , where *t_H_* is the time needed for locating hash entries, and *P* is a pattern to be inserted into the set of maximal patterns [24].

**Figure 1:**
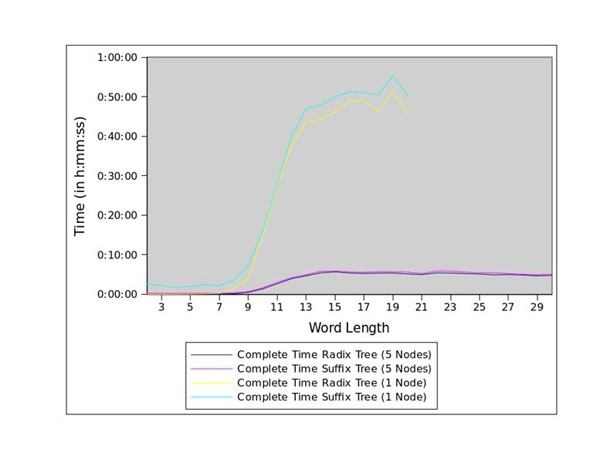
Complete run-times for the core promoters of *Arabidopsis thaliana*

While a number of algorithms and software tools have been developed to solve the word discovery problem, most do not provide the scalability needed to process large (genome-scale) data sets. For example, our single-processor enumeration methods, based on either a radix tree or a suffix tree, are unable to perform word enumeration for the ~27,000 core promoters of the *Arabidopsis thaliana* genome for word lengths greater than 19bp (see Figure 1).

The WordSeeker software suite addresses this problem by providing scalable word discovery algorithms. The software described herein builds upon earlier work of the authors (reported in [32]), which developed cache aware data layout and access strategies for a shared memory implementation of the radix tree data structure. WordSeeker has been used to analyze the promoter regions of genes in the DNA repair pathways of *Homo sapiens*[25], the entire genome of *Arabidopsis thaliana*[26], and regulatory regions involved in gravity response in *Arabidopsis thaliana*[27]. As reported in [28], results of the WordSeeker analysis of the *Arabidopsis thaliana* genome have been incorporated into AGRIS - the Arabidopsis Gene Regulatory Information Server [29].

The remainder of the manuscript presents a description of the methods employed by WordSeeker, an experimental assessment of their effectiveness, and a discussion of results.

## Methods

This section presents the software design, the concurrent architecture, the open source repository and the deployment guidelines for the WordSeeker software.

### Software architecture

The Open Word Enumeration Framework (OWEF) [30,31] provides the ability to employ different motif discovery algorithms without changing the overall execution logic of the software system. For example, WordSeeker can utilize a radix tree or a suffix tree for word space enumeration. This enables the selection of the “best” algorithm for a specific dataset at run-time, as necessitated by input parameters and dataset characteristics. For example, it is recommended that the suffix tree be used when enumerating long words (>24bp) and that the radix tree be used when enumerating short words.

The OWEF controls a set of classes responsible for specific functions. A set of input sequences is processed by a word enumeration algorithm, which store the words in a data structure. The stored information structure is processed by the *WordScoring* function to form a statistical model. The model, and more importantly operations on the model, are provided to other classes via *OWEFArgs*. Other classes, such as *SequenceClustering, WordDistribution, Cluster, ModuleDiscovery* and *WordFamily*, use the information to identify statistically significant words, which are used to discover motifs, modules, and sequence clusters.

### Distributed architecture

**Figure 2:**
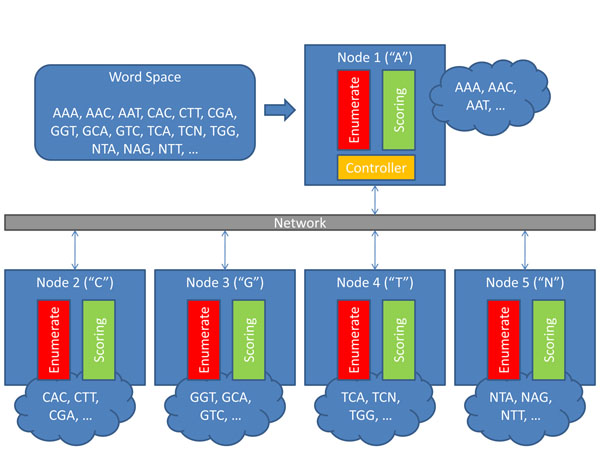
Distributed architecture of WordSeeker

WordSeeker uses a two-level parallelization strategy to achieve scalability with respect to input parameters, and with respect to the numbers of cluster nodes and processor cores. Node-level parallelization (Figure 2) uses the message passing interface (MPI) for coordination and communication between nodes. A controller task coordinates the activities of worker nodes. During the *word enumeration phase*, the data structure representing the word space (e.g., the radix tree or the suffix tree) is distributed to worker nodes. Data partitioning is accomplished by creating a list of prefixes for each worker node (as described in [32] and [33]). Thus, each node builds a portion of the overall data structure.

During the *word scoring phase*, loop-level parallelism is exploited by partitioning statistical analysis among the cores of the worker nodes, each of which utilizes a distributed Markov chain model for the computation of scores for a subset of the enumerated words. During word scoring, nodes share word occurrence information as needed. OpenMP compiler directives are used to define parallel sections and to add parallel loop constructs. This allows automatic generation of multi-threaded code, if the target compiler supports OpenMP extensions. (If OpenMP support is not available, the directives are simply ignored.)

### Open Source implementation

WordSeeker was developed in the Ohio University Bioinformatics Laboratory on a 5-node cluster computer. Each node contains 32GB RAM, 8 cores, 2TB hard disk space (a RAID5 array) and a dual-channel, gigabit ethernet.

The public version of WordSeeker, which can be accessed at http://word-seeker.org, is deployed on the Ohio Supercomputer Center’s Glenn cluster, an IBM e1350 system with more than 4200 Opteron processor cores that are connected by 10 Gbps or 20 Gbps Infiniband. WordSeeker ‘jobs’ are started and controlled through the Ohio Supercomputer Center’s job management system. The porting of the WordSeeker software from the Ohio University cluster computer to the Glenn cluster was easily accomplished, by observing the open source policies that are highlighted in this section (and detailed in the WordSeeker open source repository).

The WordSeeker source code, released under GNU General Public License v3, is available at http://code.google.com/p/word-seeker/. Access to the source code can be achieved through svn at http://word-seeker.googlecode.com/svn/trunk. The source code is documented using the doxygen code generator.

To build an executable version of WordSeeker, the C++ compiler version, 4.1* or higher is required, as well as OpenMP headers. The distributed version of WordSeeker requires a working MPI environment with MPICH2, MPIEXEC and MPICXX installed. The visualization capabilities require Perl 5.8.8, the Perl TFBS module (http://tfbs.genereg.net/) and gnuplot, version 4.2 or higher. WordSeeker has been tested under Ubuntu 9.04 and the linux operating system provided in the Ohio Supercomputer Center environment.

## Results and discussion

This section presents results of a comprehensive suite of tests performed to evaluate the performance and scalability of the different parallel and distributed modes of WordSeeker. Specifically, the evaluations considered the single-node version, the OpenMP-based shared-memory multiprocessors / multicore version, the MPI-based distributed (multiple node cluster) version, and a mixed shared-memory/distributed memory version. Shared memory tests were performed on a 64-bit Linux machine with 4 Dual-Core 2.6 gigahertz AMD Opteron processors and 32 GB of RAM. Distributed memory tests were performed on a 64-bit Linux machine with 4 Quad-Core 2.5 gigahertz AMD Opteron processors and 24 GB of RAM.

WordSeeker was evaluated under diverse circumstances by varying (1) the size of the input DNA sequence, (2) the length of DNA words to be analyzed, and (3) the enumeration algorithm (a radix tree and a suffix tree were used). The evaluation involved the measurement of (1) *computational performance* - the overall execution time of the software, and the execution times for specific functions; (2) *speed-up* - the sequential execution time divided by the parallel execution time; and (3) *efficiency* - speed-up divided by the total number of nodes (or cores) used.

## Performance

A set of experiments analyzes the overall performance of the WordSeeker pipeline for the core promoters of the *Arabidopsis thaliana* genome (for a detailed characterization of the *Arabidopsis thaliana* genome using WordSeeker see [26]). The tests compare the single core version and the distributed version. The core promoters include 100 nucleotides directly upstream of 27,167 transcription start sites. To determine the relationship between word length and performance, the complete run-times, as well as the run-times for the enumeration and the scoring stages, were computed for word lengths in the range [2bp, 30bp]. The rationale for choosing this range of word lengths is as follows. While eukaryotic transcription factors usually recognize 6-8bp long binding sites [34,35], much longer functional binding sites have been discovered (e.g., AGRIS [29] describes a 29bp binding site).

**Figure 3:**
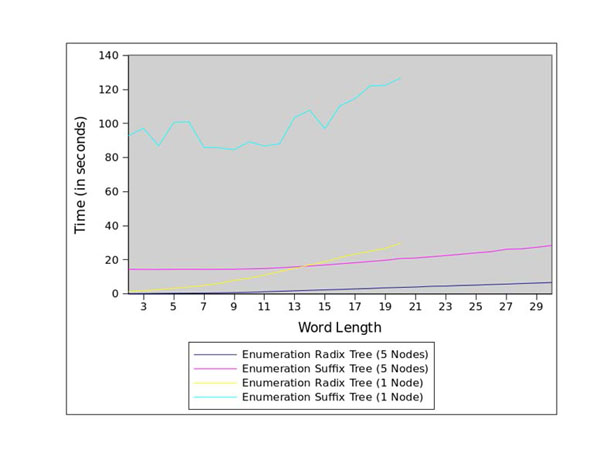
Enumeration run-times for the core promoters of *Arabidopsis thaliana*

**Figure 4:**
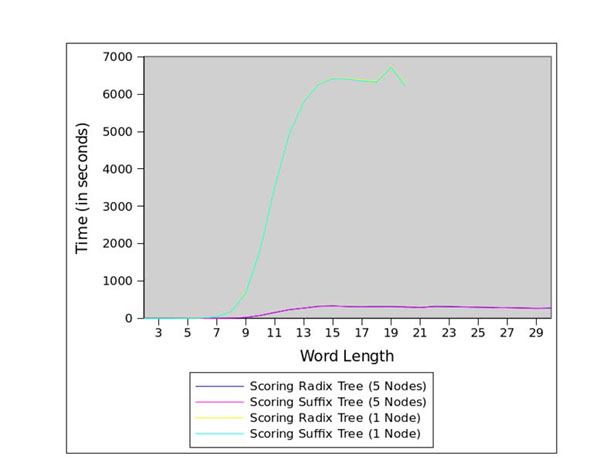
Scoring run-times for the core promoters of *Arabidopsis thaliana*

Figure 1 presents the total run-time, while Figures 3 and 4 present, respectively, the run-times for the enumeration stage and the scoring stage. While the sequential version and the distributed version exhibit similar run-times for word lengths less than 7bp, the run-time performance of the sequential version decreases significantly for larger word lengths. Due to the exhaustion of available memory in the single-node version, the sequential analysis *cannot run* for word lengths greater than 19bp. The concurrent versions were able to run for the entire [2bp, 30bp] range.

**Figure 5:**
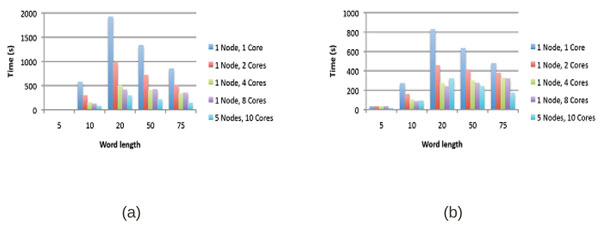
Mixed distributed/shared memory results for the core promoters of *Arabidopsis thaliana* using the Radix Tree (a) and Suffix Tree (b) data structures

Figures 5a and 5b compare the performance results for a multi-threaded version of WordSeeker, which used (1) a single computing node and (2) five computing nodes. The single node version utilizes 1, 2, 4, and 8 cores, and the five node version uses 2 cores/node, for a total of 10 cores. The plots of the overall execution times for the various word lengths demonstrate that the concurrent algorithms provide scalability by effectively utilizing the distributed hardware.

## Speedup and efficiency

**Figure 6:**
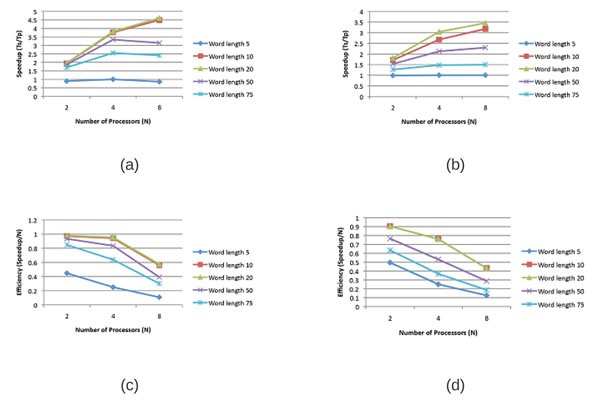
Shared memory speedups for Radix Tree (a) and Suffix Tree (b) implementations as well as shared memory efficiencies for Radix Tree (c) and Suffix Tree (d) implementations

Speedup and efficiency experiments were performed to assess in detail the scalability and the performance boundaries of the WordSeeker implementation. Figures 6a and 6b show the speedup, and Figures 6c and 6d show the efficiency, of shared memory implementations of the radix tree and the suffix tree on 2, 4, and 6 processor cores.

The speedup and efficiency results show a drop in performance for very short words (5bp) and for very long words, (50bp and 75bp), but yield good results for word lengths of 10bp and 20bp. The performance drop for short word lengths occurs because the parallelization overhead outweighs the computational benefit; for longer word lengths, cache inefficiency and front-side bus contention cause performance to decrease (see [33] for a detailed analysis of caching effects in this context). The suffix tree performed similarly to the radix tree in terms of speedup and efficiency. The difference between Figures 6c and 6d can be attributed to the cost of suffix tree construction.

## Conclusions

WordSeeker is a general purpose, scalable, open source approach to word enumeration. It supports an important set of use cases, has been applied to interesting case studies, and effectively exploits parallel and distributed computing hardware to provide scalable performance.

WordSeeker is being used currently to perform complete word space enumerations on a genomic scale; to construct word and motif encyclopedias for whole genomes; to perform word-based characterizations of pathways, tissues, and co-regulated genes; and to identify motifs in ChIP-Seq data. Ongoing work includes the construction of OpenMotif, a project that combines a number of motif discovery open source projects into a cohesive framework.

## List of abbreviations used

ENCODE: Encyclopedia of DNA Elements; DNA: Deoxyribonucleic acid; AGRIS: The Arabidopsis Gene Regulatory Information Server; UTR: Untranslated Region; ChIP-chip: Chromatin Immunoprecipitation with microarray technology; ChIP-Seq: Chromatin Immunoprecipitation with massively parallel DNA sequencing; OWEF: Open Word Enumeration Framework; MPI: Message Passing Interface; A, C, G, T: Adenine, Cytosine, Guanine, Thymine; RAID: Redundant Array of Independent Disks.

## Competing interests

The authors declare that they have no competing interests.

## Authors' contributions

JL contributed to the design, implementation and validation of the algorithms and models, the generation of the results and the writing of this document. KK, LN, LJN contributed to the development and implementation of the models and algorithms and the generation of the results. XL, RA contributed to the generation of the results. JDW, EJ and TB contributed to the development and implementation of the models and algorithms. KE and SSL contributed to the development of the models and algorithms. LE contributed to the development of the biological models. In addition to conceptualizing the architecture employed in this research, FD and LRW contributed to the design and validation of models and algorithms, and to the writing of this manuscript.

## References

[B1] The ENCODE Project Consortium The ENCODE (ENCyclopedia Of DNA Elements) Project Science 2004 306 636 640 10.1126/science.1105136 15499007

[B2] Blahnik KR Dou L O'Geen H McPhillips T Xu X Cao AR Iyengar S Nicolet CM Ludascher B Korf I Farnham PJ Sole-Search: an integrated analysis program for peak detection and functional annotation using ChIP-seq data Nucl Acids Res 2010 38 3 e13 10.1093/nar/gkp1012 19906703 PMC2817454

[B3] Feng J Naiman DQ Cooper B Coding DNA repeated throughout intergenic regions of the Arabidopsis thaliana genome: evolutionary footprints of RNA silencing Molecular BioSystems 2009 5 1679 1687 10.1039/b903031j 19452047

[B4] Rigoutsos I Huynh T Miranda K Tsirigos A McHardy A Platt D Short blocks from the noncoding parts of the human genome have instances within nearly all known genes and relate to biological processes Proc Natl Acad Sci U S A 2006 103 6605 6610 10.1073/pnas.0601688103 16636294 PMC1447521

[B5] Heath L Pati A Mandoiu I, Zelikovsky A Genomic Signatures from DNA Word Graphs Bioinformatics Research and Applications 2007 Springer Berlin/Heidelberg 317 328 Lecture Notes in Computer Science, vol 4463full_text

[B6] Blanchette M Sinha S Separating real motifs from their artifacts Bioinformatics 2001 17 S30 38 11472990 10.1093/bioinformatics/17.suppl_1.s30

[B7] Fauteux F Blanchette M Stromvik MV Seeder: discriminative seeding DNA motif discovery Bioinformatics 2008 24 2303 2307 10.1093/bioinformatics/btn444 18718942 PMC2562012

[B8] Hoebeke M Schbath S R'MES: Finding Exceptional Motifs, version 3 User Guide 2006 L'institut nationl de la recherché agronomique;

[B9] Prum B Rodolphe F Turckheim Ed Finding Words with Unexpected Frequencies in Deoxyribonucleic Acid Sequences Journal of the Royal Statistical Society Series B (Methodological) 1995 57 205 220

[B10] Sagot M-F Lucchesi C, Moura A Spelling Approximate Repeated or Common Motifs Using a Suffix Tree LATIN'98: Theoretical Informatics 1998 Springer: Berlin/Heidelberg 374 390 Lecture Notes in Computer Science vol 1380full_text

[B11] Schbath S Prum B de Turckheim E Exceptional motifs in different Markov chain models for a statistical analysis of DNA sequences J Comput Biol 1995 2 417 437 10.1089/cmb.1995.2.417 8521272

[B12] Sharma D Rajasekaran S A Simple Algorithm for (l, d) Motif Search Proceedings of the 6th Annual IEEE conference on Computational Intelligence in Bioinformatics and Computational Biology 2009 IEEE Press: Piscataway 148 154 30 March-02 April 2009; Nashville

[B13] Sinha S Tompa M Russ Altman, Timothy L. Bailey, Philip Bourne, Michael Gribskov, Thomas Lengauer, Ilya N. Shindyalov, Lynn F.Ten Eyck, and Helge Weissig A statistical method for finding transcription factor binding sites Proceedings of the Eighth International Conference on Intelligent Systems for Molecular Biology: 19–23 August 2000; La Jolla 2000 The AAAI Press, Menlo Park 344 354 10977095

[B14] Sinha S Tompa M YMF: a program for discovery of novel transcription factor binding sites by statistical overrepresentation Nucl Acids Res 2003 31 3586 3588 10.1093/nar/gkg618 12824371 PMC169024

[B15] Wang G Yu T Zhang W WordSpy: identifying transcription factor binding motifs by building a dictionary and learning a grammar Nucl Acids Res 2005 33 W412 416 10.1093/nar/gki492 15980501 PMC1160252

[B16] Wang G Zhang W A steganalysis-based approach to comprehensive identification and characterization of functional regulatory elements Genome Biol 2006 7 R49 10.1186/gb-2006-7-6-r49 16787547 PMC1779545

[B17] Pavesi G Mereghetti P Mauri G Pesole G Weeder Web: discovery of transcription factor binding sites in a set of sequences from co-regulated genes Nucl Acids Res 2004 32 W199 203 10.1093/nar/gkh465 15215380 PMC441603

[B18] Kurtz S Choudhuri JV Ohlebusch E Schleiermacher C Stoye J Giegerich R REPuter: the manifold applications of repeat analysis on a genomic scale Nucl Acids Res 2001 29 4633 4642 10.1093/nar/29.22.4633 11713313 PMC92531

[B19] Apostolico A Bock ME Lonardi S Xu X Efficient detection of unusual words J Comput Biol 2000 7 71 94 10.1089/10665270050081397 10890389

[B20] Pevzner PA Sze SH Russ Altman, Timothy L. Bailey, Philip Bourne, Michael Gribskov, Thomas Lengauer, Ilya N.Shindyalov, Lynn F.TenEyck, and Helge Weissig Combinatorial approaches to finding subtle signals in DNA sequences Proceedings of the Eighth International Conference on Intelligent Systems for Molecular Biology: 19–23 August 2000; La Jolla 2000 The AAAI Press, Menlo Park 269 278 10977088

[B21] Eskin E Pevzner PA Finding composite regulatory patterns in DNA sequences Bioinformatics 2002 18 S354 363 12169566 10.1093/bioinformatics/18.suppl_1.s354

[B22] Rigoutsos I Floratos A Combinatorial pattern discovery in biological sequences: The TEIRESIAS algorithm Bioinformatics 1998 14 55 67 10.1093/bioinformatics/14.1.55 9520502

[B23] Karp RM Miller RE, Thatcher JW Reducibility Among Combinatorial Problems Complexity of Computer Computations 1972 New York: Plenum 85 103

[B24] Floratos A Rigoutsos I On the Time Complexity of the TEIRESIAS Algorithm Research Report 1998 IBM T.J. Watson Research Center

[B25] Lichtenberg J Jacox E Welch J Kurz K Liang X Yang M Drews F Ecker K Lee S Elnitski L Welch L Word-based characterization of promoters involved in human DNA repair pathways BMC Genomics 2009 10 Suppl 1 S18 10.1186/1471-2164-10-S1-S18 19594877 PMC2709261

[B26] Lichtenberg J Yilmaz A Welch J Kurz K Liang X Drews F Ecker K Lee S Geisler M Grotewold E Welch L The word landscape of the non-coding segments of the Arabidopsis thaliana genome BMC Genomics 2009 10 463 10.1186/1471-2164-10-463 19814816 PMC2770528

[B27] Liang X Shen K Lichtenberg J Wyatt SE Welch LR An integrated bioinformatics approach to the discovery of *cis*-regulatory elements involved in plant gravitropic signal transduction International Journal of Computational Bioscience 2010 1 1 33 54

[B28] Lichtenberg J Yilmaz A Kurz K Liang X Nelson C Bitterman T Stockinger E Grotewold E Welch LR Elnitski L, Piontkivska H, Welch L Encyclopedias of DNA elements for Plant Genomes Advances in Genomic Sequence Analysis and Pattern Discovery 2011 Hackensack: World Scientific Publishing Company; (in press)

[B29] Davaluri RV Sun H Palaniswamy SK Matthews N Molina C Kurtz M Grotewold E AGRIS Arabidopsis Gene Regulatory Information Server, an information resource of Arabidopsis cis-regulatory elements and transcription factors BMC Bioinformatics 2003 4 1 25 10.1186/1471-2105-4-25 12820902 PMC166152

[B30] Kurz K Lichtenberg J Nau L Drews F Welch LR An Open Source Framework for Bioinformatics Word Enumeration and Scoring10th Annual Bioinformatics Open Source Conference BOSC: 27-28 June 2009; Stockholm 2009 37

[B31] Kurz K A Parallel, High-Throughput Framework for Discovery of DNA Motifs 2010 Ohio University Electrical Engineering and Computer Science

[B32] Tian Y Tata S Hankins RA Patel JM Practical methods for constructing suffix trees The VLDB Journal 2005 14 3 281 299 10.1007/s00778-005-0154-8

[B33] Drews F Lichtenberg J Welch L Scalable parallel word search in multicore/multiprocessor systems J Supercomput 2010 51 58 75 10.1007/s11227-009-0308-3

[B34] Tompa M Li N Bailey TL Church GM De Moor B Eskin E Favorov AV Frith MC Fu Y Kent WJ Assessing computational tools for the discovery of transcription factor binding sites Nature Biotechnology 2005 23 137 144 10.1038/nbt1053 15637633

[B35] Grotewold E Springer N The Plant Genome: Decoding the Transcriptional Hardwiring Annual Plant Reviews 2009 35 196 227

